# Case Report: Single visit photo-activated disinfection in regenerative endodontics

**DOI:** 10.12688/f1000research.20118.2

**Published:** 2020-06-03

**Authors:** Al-Shaimaa Abdel Hafiz Abdel Rahim, Fatma Abdelgawad, Ahmed M. Abd Alsamed, Dalia Mohamed Moheb, Norhan Abdel Wahab El-Dokky

**Affiliations:** 1 Pediatric Dentistry and Dental Public Health Department, Faculty of Dentistry, Fayoum University, Fayoum, 63514, Egypt; 2 Pediatric Dentistry and Dental Public Health Department, Faculty of Dentistry, Cairo University, Cairo, 11553, Egypt; 3 Oral and Maxillofacial Radiology Department, Faculty of Dentistry, Cairo University, Cairo, 11553, Egypt

**Keywords:** regenerative endodontics, necrotic tooth, open apex, root canal disinfection

## Abstract

**Background:** Root canal disinfection is considered critical for achieving successful regenerative endodontic procedures. Photo-activated disinfection is a novel disinfection method that can help to achieve the goal of regenerative endodontics. This article reports the clinical and radiographic results after single visit regenerative endodontics using photo-activated disinfection.

**Methods:** An 8.5-year-old girl complained of fractured upper right central incisor. Pulp necrosis was diagnosed on the basis of clinical findings. The root canal was irrigated with sodium hypochlorite solution (1.5%) followed by saline. Then, the canal was dried with paper points. A combination of a photosensitizer solution and low power laser light were applied. EDTA solution was used as a final irrigant. Bleeding was induced, followed by placement of collagen resorbable matrix and white mineral trioxide aggregate. Two days later, the tooth was sealed and restored with permanent filling.

**Results:** Clinical findings revealed no pain on percussion or palpation tests. Radiographic examination revealed an increase in root length, an increase of apical root thickness and apical closure at the 12-month follow-up period.

**Conclusion:** Regenerative endodontics using photo-activated disinfection achieved successful outcomes in the necrotic immature permanent tooth.

## Introduction

The term regenerative endodontic procedures (REPs) has been widely endorsed. This term describes all procedures that aim to achieve organized repair of the dental pulp ^1^. The clinical considerations for REPs include: disinfecting the root canal system; providing a scaffold with periapical tissue laceration to get a blood clot and introduce stem cell activity within the root canal; and sealing the coronal access properly to prevent reinfection ^2, 3^.

Antibiotics appear to be suitable intra-canal medication. Triple antibiotic paste (TAP) consisting of metronidazole, ciprofloxacin and minocycline is considered to be a successful regimen in managing the root canal pathogen of necrotic immature permanent teeth ^4^. Recently, new methods of disinfection have been described to control the limitations of conventional disinfecting methods by neither diminishing the bacterial number to an adequate level or eliminating the toxicity to periapical stem/progenitors ^5^. Other concerns regarding the use of TAP include tooth discoloration and bacterial resistance ^6^.

Photodynamic therapy (PDT)/photo-activated disinfection (PAD) is considered one of the optimized single visit approaches ^7^. It involves a photosensitizer (photoactive dye) that is activated by exposure to light of a specific wavelength in the presence of oxygen. The energy transferred from the activated photosensitizer to available oxygen leads to toxic oxygen species formation, such as singlet oxygen and free radicals. These very reactive chemical species can destroy proteins, nucleic acids, lipids and other cellular components ^8^. Moreover, PAD acts in a selective way. Both photosensitizer and oxygen released during bacterial cell death do not exhibit toxicity to the viable tissues ^9^.

Several *in vitro* studies have shown that PDT is effective in root canal disinfection ^10– 12^. A clinical study by Johns *et al.* ^13^ reported successful outcomes for using PAD in root canal treatment of completely formed root. The case report by Johns *et al.* ^14^ was the first to document pulp revascularization using PDT and platelet-rich fibrin. Root lengthening, continued thickening of the canal walls and apical closure were demonstrated at 10-month follow-up ^14^.

This article reports the successful use of PAD in regenerative endodontics as a novel and effective disinfection method, which might present a solution to the problems associated with triple antibiotic paste ^14^.

## Case report

**Figure 1.**
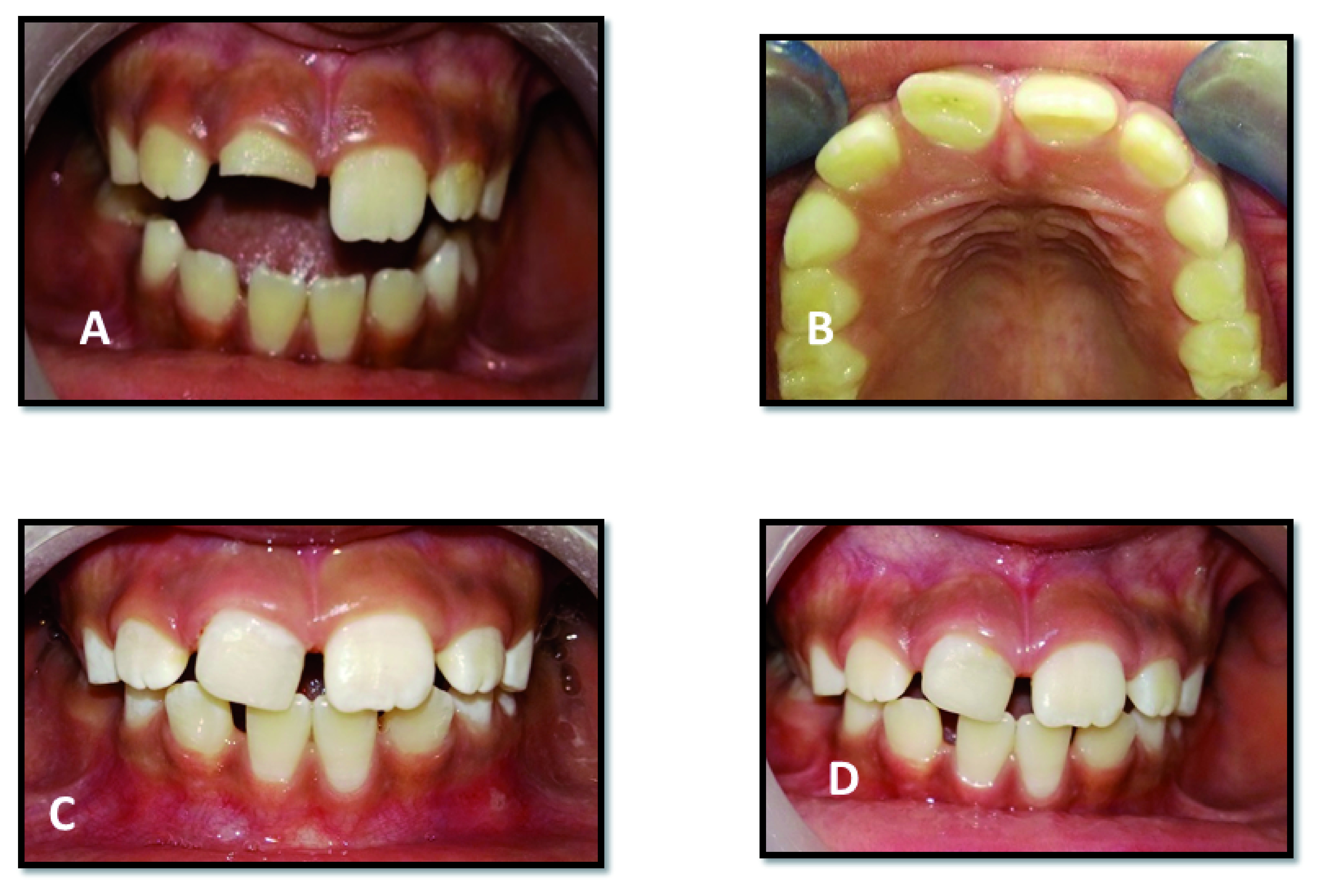
**A**) front view of preoperative intra-oral photo of traumatized upper right central incisor; **B**) occlusal preoperative photo; **C**) three month follow up; and **D**) 12-month follow-up.

**Figure 2.**
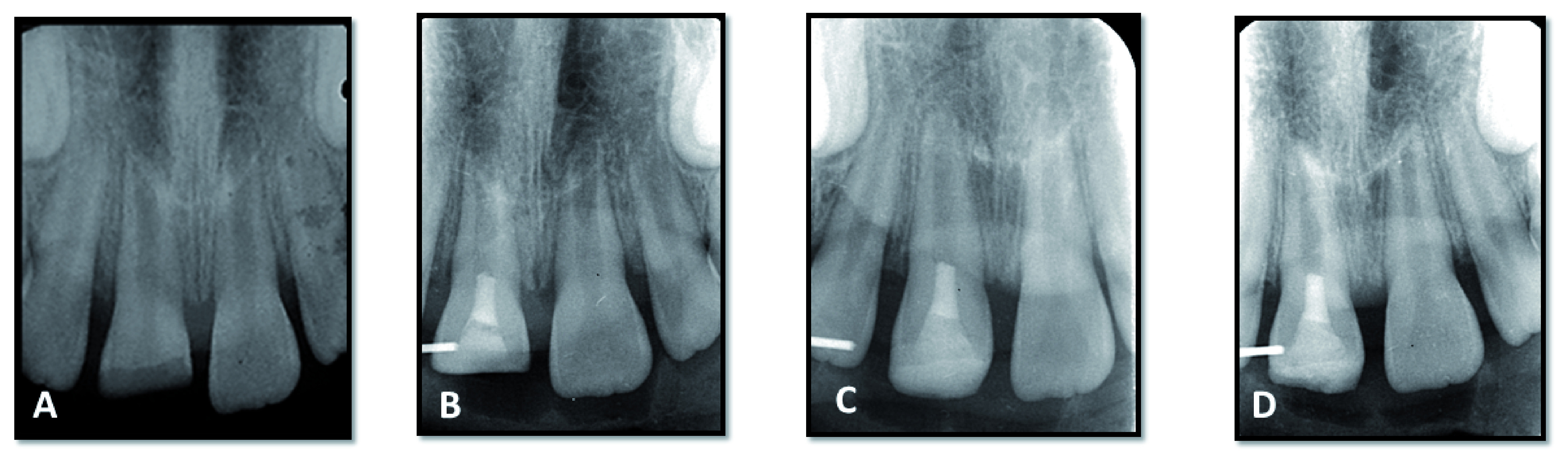
**A**) preoperative periapical radiograph; **B**) immediate postoperative radiograph after mineral trioxide aggregate coronal plug application; **C**) follow up radiograph at six months; and **D**) follow-up radiograph at 12 months.

An 8.5-year-old Egyptian girl came to the outpatient clinic of Pediatric Dentistry and Dental Public Health Department, Faculty of Dentistry, Cairo University with the chief complaint of fractured upper right central incisor due to trauma one and half months previously ( Figure 1A and B). The patient’s medical history was non-contributory. On clinical examination (which included a visual examination for any abnormalities, palpation of labial vestibule, percussion test and sensibility test), the tooth was sensitive to percussion, which was determined by tapping the tooth with the back of the mirror. The surrounding soft tissue had no tenderness to finger palpation and the tooth had no response to the hot test in comparison to the contralateral tooth. Preoperative radiographic examination revealed a wide root canal with an open apex ( Figure 2A) using conventional periapical radiograph.

The case was managed by single visit regenerative endodontic procedure using PAD after obtaining written informed consent from the parents (including usage of data and publication) and assent from the child. The procedure was performed according to the American Association of Endodontics (AAE) guidelines ^15^, except PAD instead of TAP was used. A 1.8ml carpule of mepivacaine 3% (Mepivacaine hydrochloride, Alexandria Co. for Pharmaceuticals, Egypt) was administered by infiltration. A rubber dam was applied, followed by access cavity preparation and tooth length detection. Gentle, copious irrigation was done using 20ml NaOCl (1.5% NaOCl 20ml/canal for five minutes). NaOCl of lower concentration was advised and then flushed with saline (20ml/canal for minutes), using irrigating needle located about 1mm from root end then the canal was dried using paper points.

Aseptim solution (SciCan Ltd, Denfotex Light Systems Ltd, Inverkeithing, Scotland) was applied to the canal, followed by a low power laser diode red light system (Aseptim system, SciCan Ltd, Denfotex Light Systems Ltd, Inverkeithing, Scotland) with a specific wavelength (635nm) to activate the aseptim solution for 150 seconds. Canals was washed with saline to remove the aseptim solution. Gentle, copious, irrigation with 20ml of 17% EDTA was applied. Dryness with paper points to remove excess EDTA was done. Bleeding was initiated into the canal system by rotating a K-file at 2mm beyond the apical foramen. A resorbable matrix (Collacote dressing Zimmer Biomet, USA) was placed over the blood clot. Then, white mineral trioxide aggregate (MTA) (Angelus, Brazil) was placed over the matrix with placement of a moistened pellet of cotton and glass ionomer ( Figure 2B). After two days, the tooth was double sealed using glass ionomer cement (Kromoglass 2, LASCOD- Italy) and composite restoration 3M composite (3M, America Inc).

Clinical examination of the patient revealed no adverse signs and symptoms at three, six-, nine- and 12-month follow-up periods ( Figure 1C and 1D). Radiographic examination revealed an increase in root length and root thickness at six months ( Figure 2C) and complete root closure at 12 months ( Figure 2D) using digital radiography (Digora™ Optime UV) and digital software (Soredex, Finland). An individual acrylic XCP (Extension Cone Paralleling) index was prepared by registering the bite and placed around the XCP plastic tip for radiographic standardization during follow up ( Table 1)

**Table 1.** The patient’s timeline of symptoms, treatment and follow-up periods.

Timeline	Event	Findings
0	Patient comes to the clinic. Patient’s medical history obtained. Clinical and radiographic examination performed.	Fractured necrotic traumatized upper right central incisor, sensitive to percussion and no response to hot test with a wide root canal and an open apex
0	Regenerative endodontic procedure using photo-activated oral disinfection	
+ 2 days	Composite restoration	
+ 3 months	1 ^st^ Follow up (clinical assessment)	No pain, no swelling (symptom free)
+ 6 months	2 ^nd^ Follow up (clinical and radiographic assessment)	No pain, no swelling (symptom free) Increase in root length and dentin root thickness, and no adverse radiographic evidence.
+ 9 months	3 ^rd^ follow up (clinical assessment)	No pain, no swelling (symptom free).
+ 12 months	4 ^th^ follow up (clinical and radiographic assessment)	No pain, no swelling (symptom free). Complete apical root closure

## Discussion

This case report documents the successful application of a novel and innovative disinfection technique in regenerative endodontics for management of a necrotic immature permanent tooth in a single visit, although a longer follow up period is recommended.

Root canal system disinfection is an integral step in the success of REPs. Chemical disinfection of the root canal is dependent on bacteriostatic/bactericidal properties of the agents as well as avoid harming the patient’s stem cells ^3^. Copious and gentle irrigation was done using 20ml NaOCl. The use of NaOCl at lower concentrations was advised (1.5% NaOCl) with the irrigating needle adjusted to be 1mm from the root end to reduce the cytotoxicity to stem cells and to minimize the possibility of irrigant extrusion into the periapical tissues ^15^. Furthermore, 1.5% NaOCl was effective in bacterial reduction of root canals as reported by Trevino *et al.* ^16^.

The PAD technique was effective in removing high bacterial concentrations from infected root canals ^17^. Aseptim solution was applied to the canal and agitated in the canal for 60 seconds using an endodontic file to ensure maximum penetration of the dye, since it is essential that the aseptim solution comes in close contact with the bacteria, otherwise the photosensitivity process does not occur ^18^. The photosensitizer stains the bacterial cells in soft and hard tissues and the photo-activated cells release molecular oxygen causing disruption of the bacterial cell wall. Photosensitizer and oxygen released during bacterial cell death are not reported to produce any toxicity to normal cells ^9^. Moreover, PAD is a newer antimicrobial strategy that involves the combination of a non-toxic PS or dyes and a non-harmful visible light source to disinfect the root canal. Low power laser in itself is not particularly lethal to bacteria, but is useful for photochemical activation of oxygen-releasing dyes ^19^.

Dickers *et al.* ^20^ stated that the average temperature rise was lower than the 7 degrees C safety level for periodontal injury. Moura-Netto *et al.* ^21^ reported that studies on tissue engineering using stem cells from human exfoliated deciduous teeth have yielded promising results. Laser phototherapy is able to influence the proliferation and differentiation of these cells

Blood clot formation inside the canal is considered a scaffold and a source of growth factors. Inducing bleeding to promote blood clot formation is a commonly used method in many of the reported cases ^22– 25^. A resorbable matrix (collagen wound dressing) should be carefully placed on the top of the blood clot to serve as an internal matrix and ease the placement of MTA ^15^. White MTA was applied to act as a coronal plug and achieve effective coronal seal. It was the most frequently chosen material in published regenerative endodontic studies ^26– 29^.

Regenerative endodontic procedures were completed during a single visit because PAD is considered one of the optimized single visit disinfection approaches. A successful single-visit regenerative endodontic therapy of an immature permanent tooth with a chronic apical abscess was published by Shin et al. They stated that a single-visit revascularization procedure has some advantages. It eliminates subsequent appointments to access the root canal environment, thus reducing the possibility of further bacterial contamination of the root canal. It also diminishes the detrimental consequences of poor patient compliance with regular follow-up evaluation ^23^. Topçuoğlu and Topçuoğlu reported that a single-visit regenerative endodontic procedure may be a favorable treatment option for an asymptomatic immature tooth with a necrotic pulp and no periapical lesion ^30^.

No adverse clinical signs and symptoms were noted during follow up periods, which is considered as a primary goal for regenerative endodontics as reported by AAE ^15^. Apical closure and increasing apical dentin thickness were observed at the 12-month follow-up period. This case report demonstrates that PAD is a promising method for controlling infection in a single visit regenerative endodontic procedure. Further randomized clinical studies are needed to assess this disinfection technique.

## Data availability

All data underlying the results are available as part of the article and no additional source data are required.

## Consent

Written informed consent for publication of their clinical details and clinical images was obtained from the parents of the patient.
