# Supplementary material for: Case Report: Single visit photo-activated disinfection in regenerative endodontics
Source: F1000Res. 2020 Jun 3;8:1519. Originally published 2019 Aug 28. [Version 2] doi: 10.12688/f1000research.20118.2 (PMC7475962; doi:10.12688/f1000research.20118.2)
Supplement: Peer review history

# Peer review: Reviewer response for version 2

**DOI:** 10.5256/f1000research.25673.r64238

**Published:** 2020-09-04

**Authors:** Gianluca Gambarini

**Affiliations:** 1 Department of Oral and Maxillo-Facial Sciences, Sapienza University of Rome, Rome, Italy

I approve the revised manuscript in the present form.

Are enough details provided of any physical examination and diagnostic tests, treatment given and outcomes?

Partly

Is the case presented with sufficient detail to be useful for other practitioners?

Yes

Is sufficient discussion included of the importance of the findings and their relevance to future understanding of disease processes, diagnosis or treatment?

Partly

Is the background of the case’s history and progression described in sufficient detail?

Yes

Reviewer Expertise:

NA

I confirm that I have read this submission and believe that I have an appropriate level of expertise to confirm that it is of an acceptable scientific standard.

# Peer review: Reviewer response for version 2

**DOI:** 10.5256/f1000research.25673.r64237

**Published:** 2020-06-10

**Authors:** Mohamed Mokhtar Nagy

**Affiliations:** 1 Endodontic Department, Faculty of Dentistry, Ain Shams University, Cairo, Egypt

The authors addressed the required comments. The article is suitable for indexing.

Are enough details provided of any physical examination and diagnostic tests, treatment given and outcomes?

Partly

Is the case presented with sufficient detail to be useful for other practitioners?

Partly

Is sufficient discussion included of the importance of the findings and their relevance to future understanding of disease processes, diagnosis or treatment?

No

Is the background of the case’s history and progression described in sufficient detail?

Partly

Reviewer Expertise:

Endodontic regeneration

I confirm that I have read this submission and believe that I have an appropriate level of expertise to confirm that it is of an acceptable scientific standard.

# Peer review: Reviewer response for version 1

**DOI:** 10.5256/f1000research.22088.r61455

**Published:** 2020-03-30

**Authors:** Mohamed Mokhtar Nagy

**Affiliations:** 1 Endodontic Department, Faculty of Dentistry, Ain Shams University, Cairo, Egypt

The manuscript focuses upon two main issues which are single visit regeneration and photo activated disinfection.

1. The title lacks the "the single visit concept".
2. The title states "oral" disinfection, this would be better as "canal" disinfection.
3. The introduction and discussion did not include the justification of the idea of single visit regeneration.
4. The introduction and discussion did not include the possible effects of laser as heat generation and effects on stem cells.
5. The manuscript did not state radiographic standardization during follow up.
6. The discussion did not justify the supposed mechanism of root canal disinfection by laser (PAD).

Are enough details provided of any physical examination and diagnostic tests, treatment given and outcomes?

Partly

Is the case presented with sufficient detail to be useful for other practitioners?

Partly

Is sufficient discussion included of the importance of the findings and their relevance to future understanding of disease processes, diagnosis or treatment?

No

Is the background of the case’s history and progression described in sufficient detail?

Partly

Reviewer Expertise:

Endodontic regeneration

I confirm that I have read this submission and believe that I have an appropriate level of expertise to confirm that it is of an acceptable scientific standard, however I have significant reservations, as outlined above.

# Response

**Published:** 2020-04-04

**Authors:** Shaimaa Abdel Hafiz

**Affiliations:** Fayoum University, Egypt

We are pleased to submit the new version of **Case Report: Photo-activated oral disinfection in regenerative endodontics. **We appreciate the constructive criticisms of the reviewer that helped us to improve our manuscript. Herein we address their concerns. All changes appear by track changes in the revised version. Hope you find our replies satisfactory.

**Response to comments of Reviewer:**

**1. The title lacks the "the single visit concept".**

Single visit photo-activated disinfection in regenerative endodontics. The title was modified.

**2. The title states "oral" disinfection, this would be better as "canal" disinfection**

I omit the word oral because the most common terminology used in literature is Photo-activated disinfection and according to the remaining title it represents the site of disinfection applied on. So its preferable to state Photoactivated disinfection as mentioned in published literature of endodontics field **(1–5)** **.**

**3. The introduction and discussion did not include the justification of the idea of singlevisit regeneration**

***For introduction:***

Photodynamic therapy (PDT)/photo-activated disinfection (PAD) is considered one of the optimized single visit approaches **(6)** **. **This part was added to introduction section.

Several *in vitro* studies have shown that PDT is effective in root canal disinfection **(1,2,7)**. A clinical study by Johns *et al.* **(3)** reported successful outcomes for using PAD in root canal treatment of completely formed root. The case report by Johns *et al.* **(4)** was the first to document pulp revascularization using PDT and platelet-rich fibrin.  Root lengthening, continued thickening of the canal walls and apical closure were demonstrated at 10-month follow-up **(4)**.All of these mentioned studies applied the PAD system in single visit. As mentioned in introduction section.

***For Discussion:***

Regenerative endodontic procedures were completed during a single visit because PAD is considered one of the optimized single visit disinfection approaches **(6)**. A successful single-visit regenerative endodontic therapy of an immature permanent tooth with a chronic apical abscess was published by Shin et al. **(8)**, They stated that a single-visit revascularization procedure has some advantages. It eliminates subsequent appointments to access the root canal environment, thus reducing the possibility of further bacterial contamination of the root canal. It also diminishes the detrimental consequences of poor patient compliance with regular follow-up evaluation **(8)** **.** Topçuoğlu and Topçuoğlu **(9)** reported that a single-visit regenerative endodontic procedure may be a favorable treatment option for an asymptomatic immature tooth with a necrotic pulp and no periapical lesion **(9)**. This part was added to discussion section

**4. The introduction and discussion did not include the possible effects of laser as heat generation and effects on stem cells.**

***For introduction:***

Moreover, PAD acts in a selective way. Both photosensitizer and oxygen released during bacterial cell death do not exhibit toxicity to the viable tissues **(10)**. This part was added to introduction section.

***For Discussion:***

Photosensitizer and oxygen released during bacterial cell death are not reported to produce any toxicity to normal cells **(10)**.

Dickers et al. **(11)** stated that the average temperature rise was lower than the 7 degrees C safety level for periodontal injury.  Moura-Netto et al. **(12)**reported that studies on tissue engineering using stem cells from human exfoliated deciduous teeth have yielded promising results. Laser phototherapy is able to influence the proliferation and differentiation of these cells. This part was added to discussion section.

**5. The manuscript did not state radiographic standardization during follow up**

An individual acrylic XCP (Extension Cone Paralleling) index was prepared by registering the bite and placed around the XCP plastic tip for radiographic standardization during follow up. This part was added to the case report section.

**6. The discussion did not justify the supposed mechanism of root canal disinfection by laser**

**(PAD).**

**Response:**

The photosensitizer stains the bacterial cells in soft and hard tissues and the photo-activated cells release molecular oxygen causing disruption of the bacterial cell wall **(10**). Moreover, PAD is a newer antimicrobial strategy that involves the combination of a non-toxic PS or dyes and a non-harmful visible light source to disinfect the root canal. Low power laser in itself is not particularly lethal to bacteria, but is useful for photochemical activation of oxygen-releasing dyes ( **13)**. This part was added to discussion section.

**References**

1         Bergmans L., Moisiadis P., Huybrechts B., Van Meerbeek B., Quirynen M., Lambrechts P. Effect of photo-activated disinfection on endodontic pathogens ex vivo. Int Endod J 2008;41(3):227–39. Doi: 10.1111/j.1365-2591.2007.01344.x.

2         Williams Jill A., Pearson Gavin J., John Colles M. Antibacterial action of photoactivated disinfection {PAD} used on endodontic bacteria in planktonic suspension and in artificial and human root canals. J Dent 2006;34(6):363–71. Doi: 10.1016/j.jdent.2005.08.002.

3         Johns Dexton Antony, Varughese Jolly Mary, Thomas Kunjamma, Abraham Aby, James Elizabeth Prabha, Maroli Ramesh Kumar. Clinical and radiographical evaluation of the healing of large periapical lesions using triple antibiotic paste, photo activated disinfection and calcium hydroxide when used as root canal disinfectant. J Clin Exp Dent 2014;6(3). Doi: 10.4317/jced.51324.

4         Johns DextonAntony, Shivashankar VasundaraYayathi, Krishnamma Shoba, Johns Manu. Use of photoactivated disinfection and platelet-rich fibrin in regenerative Endodontics. J Conserv Dent 2014;17(5):487. Doi: 10.4103/0972-0707.139850.

5         Bonsor S. J., Nichol R., Reid T. M S, Pearson G. J. Microbiological evaluation of photo-activated disinfection in endodontics (An in vivo study). Br Dent J 2006;200(6):337–41. Doi: 10.1038/sj.bdj.4813371.

6         Siqueira José F., Rôças Isabela N. Optimising single-visit disinfection with supplementary approaches: A quest for predictability. Aust Endod J 2011. Doi: 10.1111/j.1747-4477.2011.00334.x.

7         Garcez Aguinaldo S., Ribeiro Martha S., Tegos George P., Núñez Silvia C., Jorge Antonio O C, Hamblin Michael R. Antimicrobial photodynamic therapy combined with conventional endodontic treatment to eliminate root canal biofilm infection. Lasers Surg Med 2007;39(1):59–66. Doi: 10.1002/lsm.20415.

8         Shin S. Y., Albert J. S., Mortman R. E. One step pulp revascularization treatment of an immature permanent tooth with chronic apical abscess: A case report. Int Endod J 2009:1118–26. Doi: 10.1111/j.1365-2591.2009.01633.x.

9         Topçuoğlu Gamze, Topçuoğlu Hüseyin Sinan. Regenerative Endodontic Therapy in a Single Visit Using Platelet-rich Plasma and Biodentine in Necrotic and Asymptomatic Immature Molar Teeth: A Report of 3 Cases. J Endod 2016;42(9):1344–6. Doi: 10.1016/j.joen.2016.06.005.

10       López Fernanda Ullmann, Kopper Patrícia Maria Poli, Bona Alvaro Della, Steier Liviu, de Figueiredo José Antônio Poli, Vier-Pelisser Fabiana Vieira. Effect of different irrigating solutions and photo-activated therapy for in vivo root canal treatment. Braz Dent J 2015. Doi: 10.1590/0103-6440201300154.

11       Dickers B., Lamard L., Peremans A., et al. Temperature rise during photo-activated disinfection of root canals. Lasers Med Sci 2009;24(1):81–5. Doi: 10.1007/s10103-007-0526-y.

12       MOURA-NETTO Cacio, FERREIRA Leila Soares, MARANDUBA Carlos Magno, MELLO-MOURA Anna Carolina Volpi, MARQUES Marcia Martins. Low-intensity laser phototherapy enhances the proliferation of dental pulp stem cells under nutritional deficiency. Braz Oral Res 2016;30(1). Doi: 10.1590/1807-3107BOR-2016.vol30.0080.

13       Mohan Dennis, Maruthingal Sunith, Indira Rajamani, et al. Photoactivated disinfection (PAD) of dental root canal system - An ex-vivo study. Saudi J Biol Sci 2016. Doi: 10.1016/j.sjbs.2015.01.013.

# Peer review: Reviewer response for version 1

**DOI:** 10.5256/f1000research.22088.r58468

**Published:** 2020-03-10

**Authors:** Gianluca Gambarini, Dario Di Nardo

**Affiliations:** 1 Department of Oral and Maxillo-Facial Sciences, Sapienza University of Rome, Rome, Italy

The article presents a case of apicogenesis performed by gold standard methodologies. The novelty is the use of a photoactivated disinfectant instead of a triple antibiotic paste. Since NaOCl and EDTA used are sufficient to remove all the tissues that could avoid a proper regeneration of the apex, the effectiveness of the light activated disinfectant is doubtful.

 The title of the article is on regenerative endodontics, that is a procedure that aims to regenerate dental pulp, but no evidences of regeneration are presented in the article instead of the production of new dentine, which is obtainable with a simple apicogenesis procedure and also without the use of triple antibiotic paste.

 The overall novelty of the article is doubtful and the evidences of the regeneration of pulp are not explained at all. Dentine formation and the closure of the apex are evidences that could be obtained with gold standard techniques that do not involve the use of other expensive appliances like diode lasers.

Are enough details provided of any physical examination and diagnostic tests, treatment given and outcomes?

Partly

Is the case presented with sufficient detail to be useful for other practitioners?

Yes

Is sufficient discussion included of the importance of the findings and their relevance to future understanding of disease processes, diagnosis or treatment?

Partly

Is the background of the case’s history and progression described in sufficient detail?

Yes

Reviewer Expertise:

Endodontics

We confirm that we have read this submission and believe that we have an appropriate level of expertise to state that we do not consider it to be of an acceptable scientific standard, for reasons outlined above.

# Response

**Published:** 2020-03-12

**Authors:** Shaimaa Abdel Hafiz

**Affiliations:** Fayoum University, Egypt

We appreciate the comments of the reviewer. Herein we address the concerns of the reviewer.

1- The article presents a case of apicogenesis performed by gold standard methodologies. 

The novelty is the use of a photoactivated disinfectant instead of a triple antibiotic paste. 

Since NaOCl and EDTA used are sufficient to remove all the tissues that could avoid a 

proper regeneration of the apex, the effectiveness of the light activated disinfectant 

is doubtful.

**Response:**

- Apicogenesis is a root complementation therapy performed in teeth with pulp vitality and consists in the removal of infected coronal pulp, maintenance of vital root pulp and its protection with biocompatible material (1).

Pulpal necrosis was diagnosed on the basis of clinical findings in our case report as mentioned in the methods section of abstract. So regenerative endodontic procedure was applied for necrotic tooth with open apex as recommended by American Association of Endodontists (AAE)(2).

- Naocl and EDTA may be sufficient to remove all necrotic tissues but in this case report, it was mentioned that the procedure was performed according to the American Association of Endodontics (AAE) guidelines (2), except PAD instead of TAP was used. The AAE guidelines recommend using intracanal medication in adjunctive to the irrigants.

- There are several in vitro and in vivo studies proved the effectiveness of PAD system in root canal disinfection. And this article mentioned some of them (4–9).

2- The title of the article is on regenerative endodontics, that is a procedure that aims to regenerate dental pulp, but no evidences of regeneration are presented in the article 

instead of the production of new dentine, which is obtainable with a simple apicogenesis

procedure and also without the use of triple antibiotic paste.

**Response:**

- Regenerative Endodontics is defined as a biologically-based procedure designed to predictably replace damaged, diseased or missing structures, including dentin and root structures as well as cells of the pulp-dentin complex, with living viable tissues, preferably of the same origin, that restore the normal physiologic functions of the pulp-dentin complex (10)

- The term regenerative endodontic procedures (REPs) has been widely adopted and refers to all procedures that aim to attain organized repair of the dental pulp and include future therapies yet to regenerative endodontic treatment of necrotic immature permanent teeth resulting in continued root development, increased thickness in the dentinal walls and apical closure. evolve in the field of regenerative endodontics (3).

3- The overall novelty of the article is doubtful and the evidences of the regeneration of pulpare not explained at all. 

Dentine formation and the closure of the apex are evidences that could be obtained with gold standard techniques that do not involve the use of other expensive appliances like diode lasers.

**Response:**

- Regenerative endodontic treatment of necrotic immature permanent teeth resulting in continued root development, increased thickness in the dentinal walls and apical closure as mentioned by AAE.

- AAE Clinical Considerations for a Regenerative Procedure (2) stated that the degree of success of Regenerative Endodontic Procedures is largely measured by the extent to which it is possible to attain primary, secondary, and tertiary goals: 

1. Primary goal: The elimination of symptoms and the evidence of bony healing.
2. Secondary goal: Increased root wall thickness and/or increased root length (desirable, but perhaps not essential)
3. Tertiary goal: Positive response to vitality testing (which if achieved, could indicate a more organized vital pulp tissue).

- Root canal system disinfection is an integral step in the success of REPs (2). Antimicrobial approaches to disinfect root canals have been proposed that include Photo-activated disinfection (PAD) as it offers a means of destroying bacteria remaining after using conventional irrigants in endodontic therapy (4).

References

1            Mente Johannes, Leo Meltem, Panagidis Dimos, et al. Treatment outcome of mineral trioxide aggregate in open apex teeth. J Endod 2013. Doi: 10.1016/j.joen.2012.10.007.

2            AAE. Clinical Considerations for a Regenerative Procedure (New, Revised 6-18-16). AAE 2016.

3            Diogenes Anibal, Henry Michael A., Teixeira Fabricio B., Hargreaves Kenneth M. An update on clinical regenerative endodontics. Endod Top 2013;28(1):2–23. Doi: 10.1111/etp.12040.

4            Bonsor S. J., Nichol R., Reid T. M S, Pearson G. J. Microbiological evaluation of photo-activated disinfection in endodontics (An in vivo study). Br Dent J 2006;200(6):337–41. Doi: 10.1038/sj.bdj.4813371.

5            Williams Jill A., Pearson Gavin J., John Colles M. Antibacterial action of photoactivated disinfection {PAD} used on endodontic bacteria in planktonic suspension and in artificial and human root canals. J Dent 2006;34(6):363–71. Doi: 10.1016/j.jdent.2005.08.002.

6            Garcez Aguinaldo S., Ribeiro Martha S., Tegos George P., Núñez Silvia C., Jorge Antonio O C, Hamblin Michael R. Antimicrobial photodynamic therapy combined with conventional endodontic treatment to eliminate root canal biofilm infection. Lasers Surg Med 2007;39(1):59–66. Doi: 10.1002/lsm.20415.

7            Bergmans L., Moisiadis P., Huybrechts B., Van Meerbeek B., Quirynen M., Lambrechts P. Effect of photo-activated disinfection on endodontic pathogens ex vivo. Int Endod J 2008;41(3):227–39. Doi: 10.1111/j.1365-2591.2007.01344.x.

8            Johns Dexton Antony, Varughese Jolly Mary, Thomas Kunjamma, Abraham Aby, James Elizabeth Prabha, Maroli Ramesh Kumar. Clinical and radiographical evaluation of the healing of large periapical lesions using triple antibiotic paste, photo activated disinfection and calcium hydroxide when used as root canal disinfectant. J Clin Exp Dent 2014;6(3). Doi: 10.4317/jced.51324.

9            Johns DextonAntony, Shivashankar VasundaraYayathi, Krishnamma Shoba, Johns Manu. Use of photoactivated disinfection and platelet-rich fibrin in regenerative Endodontics. J Conserv Dent 2014;17(5):487. Doi: 10.4103/0972-0707.139850.

10          Murray Peter E., Garcia-Godoy Franklin, Hargreaves Kenneth M. Regenerative Endodontics: A Review of Current Status and a Call for Action. J Endod 2007;33(4):377–90. Doi: 10.1016/j.joen.2006.09.013.
